# Prediction of Anti-Glioblastoma Drug-Decorated Nanoparticle Delivery Systems Using Molecular Descriptors and Machine Learning

**DOI:** 10.3390/ijms222111519

**Published:** 2021-10-26

**Authors:** Cristian R. Munteanu, Pablo Gutiérrez-Asorey, Manuel Blanes-Rodríguez, Ismael Hidalgo-Delgado, María de Jesús Blanco Liverio, Brais Castiñeiras Galdo, Ana B. Porto-Pazos, Marcos Gestal, Sonia Arrasate, Humbert González-Díaz

**Affiliations:** 1 Computer Science Faculty, University of A Coruna, 15071 A Coruña, Spain; pablo.gutierrez@udc.es (P.G.-A.); m.blanes@udc.es (M.B.-R.); ismael.hidalgo.delgado@udc.es (I.H.-D.); brais.cgaldo@udc.es (B.C.G.); ana.portop@udc.es (A.B.P.-P.); 2 Centro de Investigación en Tecnologías de la Información y Las Comunicaciones (CITIC), Campus de Elviña s/n, 15071 A Coruña, Spain; 3 Biomedical Research Institute of A Coruña (INIBIC), University Hospital Complex of A Coruña (CHUAC), 15006 A Coruña, Spain; 4 IKERDATA S.L., ZITEK, University of Basque Country UPVEHU, Rectorate Building, 48940 Leioa, Spain; sonia.arrasate@ehu.es (S.A.); humberto.gonzalezdiaz@ehu.es (H.G.-D.); 5 Department of Organic and Inorganic Chemistry, University of the Basque Country (UPV/EHU), 48940 Leioa, Spain; blancoliverio@gmail.com; 6 IKERBASQUE, Basque Foundation for Science, 48011 Bilbao, Spain; 7 BIOFISIKA, Basque Center for Biophysics, University of Basque Country UPVEHU, 48940 Leioa, Spain

**Keywords:** decorated nanoparticles, drug delivery, anti-glioblastoma, big data, perturbation theory, machine learning, ChEMBL database

## Abstract

The theoretical prediction of drug-decorated nanoparticles (DDNPs) has become a very important task in medical applications. For the current paper, Perturbation Theory Machine Learning (PTML) models were built to predict the probability of different pairs of drugs and nanoparticles creating DDNP complexes with anti-glioblastoma activity. PTML models use the perturbations of molecular descriptors of drugs and nanoparticles as inputs in experimental conditions. The raw dataset was obtained by mixing the nanoparticle experimental data with drug assays from the ChEMBL database. Ten types of machine learning methods have been tested. Only 41 features have been selected for 855,129 drug-nanoparticle complexes. The best model was obtained with the Bagging classifier, an ensemble meta-estimator based on 20 decision trees, with an area under the receiver operating characteristic curve (AUROC) of 0.96, and an accuracy of 87% (test subset). This model could be useful for the virtual screening of nanoparticle-drug complexes in glioblastoma. All the calculations can be reproduced with the datasets and python scripts, which are freely available as a GitHub repository from authors.

## 1. Introduction

Drug-decorated nanoparticles (DDNPs) have many medical applications, such as drug delivery systems for different types of chemical compounds [1,2]. These systems make it possible to study different combinations of drugs and nanoparticles designed to treat specific medical conditions. Nevertheless, due to the huge number of combinations, testing in a wet lab is not possible. Moreover, the synthesis of nanoparticles is expensive and time consuming, whereby computational models are useful for predicting the possible forming of effective drug-nanoparticle pairs.

According to the World Health Organization, glioblastoma multiforme (GBM) is the most common and one of the most malignant central nervous system tumors. Treatment of this cancer is still being studied due to GBM’s location in the intracranial space and the presence of the blood-brain barrier, which has particularly selective permeability to some drugs. Current treatment involves surgical intervention and the application of temozolomide and radiation, and it only guarantees a median survival of 15 months. As a result of the high invasiveness of this treatment, some research has focused on nanotechnology, which seems to be the best candidate for treating this disease, while avoiding the inconveniences of the current procedure. Some nanomaterials have been proven to pass through the blood-brain barrier and remain in GBM tissues; they could be used to co-deliver a wide variety of antitumor drugs [3,4].

In order to mix nanotechnology, chemistry, and data analysis, the PTML method was proposed by combining Perturbation Theory (PT) with Machine Learning (ML) [5,6,7,8,9,10,11,12,13,14,15,16]. Thus, different PT operators can be used to mix the original molecular descriptors with the experimental conditions in order to predict biological activity. Some PT operators are a generalization of chemoinformatics [17].

This paper mixes the perturbations of molecular descriptors of nanoparticle-drug pairs into a classifier to predict the probability of nanoparticle-drug complexes having anti-glioblastoma activity. Molecular properties, such as Polar Surface Area (PSA) and logarithmic term (logP) of the octanol/water partition coefficient (P) [18], are used as original descriptors for drugs. The logP values, such as ALogP, were calculated by approximation [19,20]. In the traditional model, the changes of the chemical structures are characterized by molecular descriptors without taking into account the variation of drug activity under different experimental conditions. Our model includes these variations of the original molecular descriptors under different experimental conditions (perturbations). Our dataset for drugs and nanoparticles was extracted from the ChEMBL database [21,22,23,24,25,26,27] and from the literature. Using the same methodology, in previous publications, we have demonstrated a similar nanoparticle-drug model against malaria [28]. The scope of this paper is to provide a free, fast, and inexpensive computational method for predicting drug-decorated nanoparticle delivery systems against glioblastoma. The model could be used to screen in silica a considerable number of possible combinations of new compounds with current or new nanoparticles (the first step in drug development). The same methodology could be extended to other specific uses of nanocarriers in different scientific fields.

## 2. Results

New PTML classification models have been constructed to predict the probability class for a nanoparticle-drug complex to have anti-glioblastoma activity. The results are important for future nanomedicine applications. The dataset for these models used mixed data from the ChEMBL database for drugs and literature sources for nanoparticles, including experimental information from pharmacological assays. Perturbation Theory (PT) was used to consider that the variation of drug-nanoparticle complexes depends on perturbations of both nanoparticle and drug properties in specific experimental conditions. Thus, the PTML models are complex functions that depend on experimental descriptors of drugs and nanoparticles as opposed to the original molecular descriptors and the mean values used in specific experimental conditions. Consequently, the models start with a probability in the dataset for each drug-nanoparticle pair and add perturbations of molecular descriptors for drugs and nanoparticles in specific experimental conditions by using moving average (MA) functions from Box-Jenkins models [29,30].

The ML methods with default parameters (for extra information, please see the GitHub repository: https://github.com/muntisa/nano-drugs-for-glioblastoma (accessed on 21 October 2021)) have generated the baseline results presented in Table 1: accuracy (ACC); area under the receiver operating characteristic curve (AUROC); precision; recall; and f1-score (using single random split of data). The best model was selected by using the AUROC and ACC metrics. Thus, the Bagging classifier is able to provide an AUROC of 0.9475 and ACC of 0.8657.

**Figure 1:**
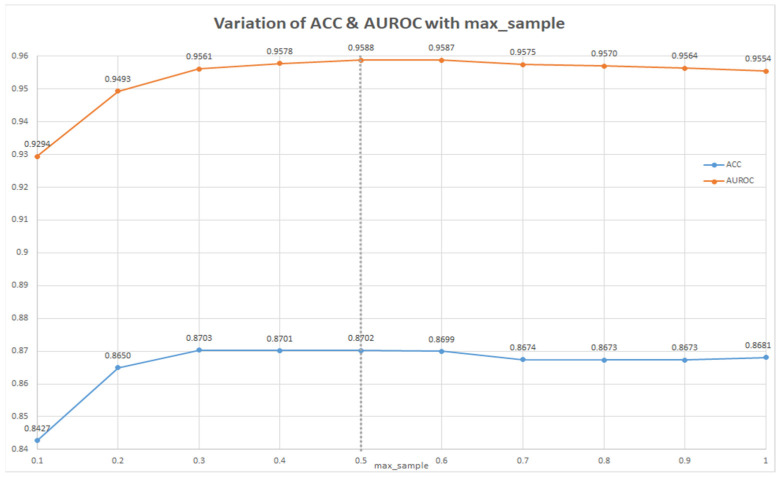
Variation of max_sample parameter for the best classifier (Bagging classifier).

**Figure 2:**
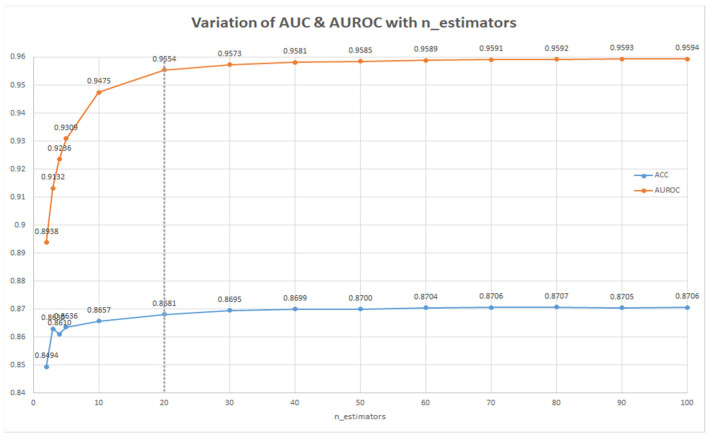
Variation of n_estimators parameter for the best classifier (Bagging classifier).

In order to improve the best baseline model, a parameter search was used. Firstly, the max_sample parameter of the Bagging classifier was tested for values from 0.1 to 1.0. The best metrics were obtained for max_sample = 0.5 (see Figure 1). Secondly, this parameter was maintained constant, and the number of decision trees was between 1 and 100 (see Figure 2). AUROC and ACC did not improve significatively from 20 to 40 trees (by doubling the number of trees) and, therefore, 20 trees was chosen as the optimal parameter. Thus, the best model for predicting nanoparticle-drug pairs with anti-glioblastoma activity was represented by the Bagging classifier with n_estimators = 20 trees and max_sample = 0.5.

## 3. Discussion

**Figure 3:**
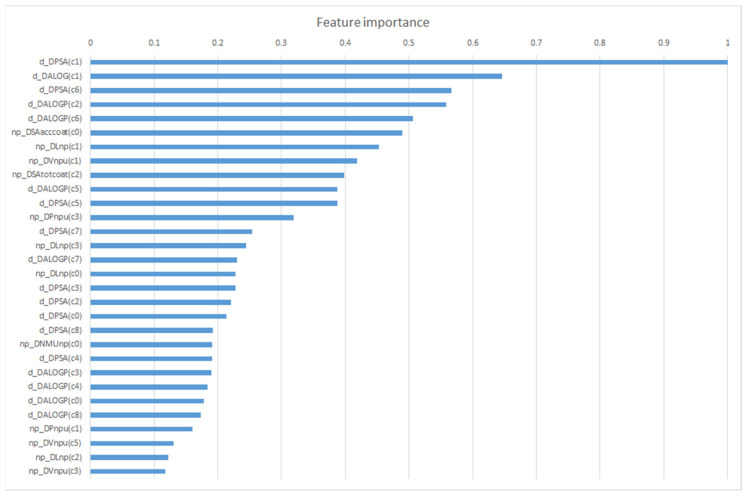
The most important features for the best classifier (normalized values).

In the next step, we studied the importance of the model features in order to understand what information is important for predicting nanoparticle-drug pairs with anti-glioblastoma activity. Thirty of the most important features for the best classifier (normalized values) are presented in Figure 3. It can be seen that both descriptors for drugs and nanoparticles are important for the classification.

The variation of PSA for drugs in different types of cells (c1) is the most important feature for this classifier, d_DPSA(c1). The polarity of the drug surface seems to be the most important feature because it is linked to the membrane solubility of the drugs. In addition, it appears that the variation of molecular descriptors for drugs and nanoparticles with the type of cells (c1) is important (see the first and most important features in Figure 3). For drugs, the perturbation of PSA seems to be more important than ALOG in different cells (c1) and organisms (c2). For nanoparticles, the most important features are (1) variations of the surface area of acceptor atoms (SAccoat); (2) np large (L) and average atomic Van der Waals volume of all atoms in the np (V) with the parameter np assay—c0(np); (3) the cell line np assay—c1(np); (4) the np shape—c2(np); and (5) np medium—c3(np) (e.g.,: np_DSAaccoat(c0), np_DLnp(c1), np_DVnpu(c1)). The most important feature for drugs was double the importance of this model compared with the most important feature for nanoparticles. Thus, the best model obtained with all features showed the experimental importance of polarity for both drugs and nanoparticles as well as the volume of nanoparticles. This analysis of feature importance is in line with the general knowledge about drug and nanoparticle properties, especially for the blood-brain barrier.

**Figure 4:**
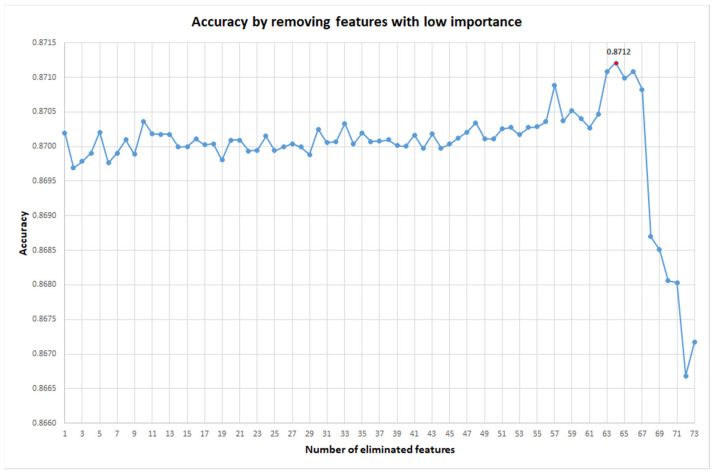
Accuracy progression with the removal of features with low importance in the best classifier.

Generally, in Machine Learning models, the least important features could add noise to the data, decreasing model performance. Therefore, we eliminated the less important features to see the course of the model’s accuracy (ACC). From the initial 104 features, we chose to eliminate 64 less important features (see Figure 4). The final model was based on only 41 features of nanoparticles and drugs: probability; d_DPSA(c0); d_DALOGP(c0); d_DPSA(c1); d_DALOG(c1); d_DPSA(c2); d_DALOGP(c2); d_DPSA(c3); d_DALOGP(c3); d_DPSA(c4); d_DALOGP(c4); d_DPSA(c5); d_DALOGP(c5); d_DPSA(c6); d_DALOGP(c6); d_DPSA(c7); d_DALOGP(c7); d_DPSA(c8); d_DALOGP(c8); np_DNMUnp(c0); np_DLnp(c0); np_DVnpu(c0); np_DPnpu(c0); np_DAMRcoat(c0); np_DSAtotcoat(c0); np_DSAacccoat(c0); np_DNMUnp(c1); np_DLnp(c1); np_DVnpu(c1); np_DPnpu(c1); np_DNMUnp(c2); np_DLnp(c2); np_DVnpu(c2); np_DPnpu(c2); np_DSAtotcoat(c2); np_DLnp(c3); np_DVnpu(c3); np_DPnpu(c3); np_DLnp(c5); np_DVnpu(c5); and np_DPnpu(c5). The final model is characterized by ACC = 0.8712, AUROC = 0.9602, precision = 0.8716, recall = 0.8712, and f1-score = 0.8714. This model can be used for future in silica screening for drug-nanoparticle pairs.

In conclusion, we demonstrated that mixing original descriptors for drugs and nanoparticles with the experimental conditions allowed us to obtain perturbations of molecular descriptors under specific conditions as inputs for classification models for the prediction of anti-glioblastoma drug-decorated nanoparticle delivery systems. The methodology tested different Machine Learning methodologies with the default parameters, improved the parameters for the best method, and reduced the number of input features using a feature selection method based on feature importance.

## 4. Materials and Methods

**Figure 5:**
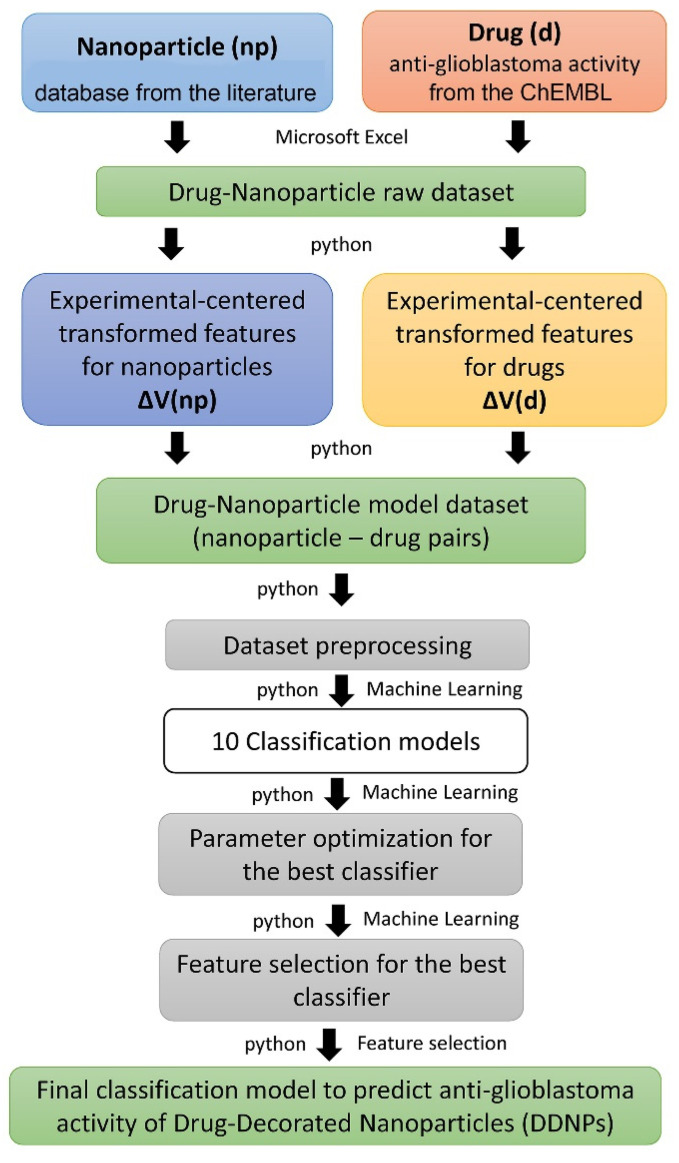
Methodology workflow for building classification models for DDNPs against anti-glioblastoma.

The proposed methodology for building classifiers for the prediction of DDNPs is based on the perturbation of molecular descriptors in specific experimental conditions (see Figure 5): (1) Raw dataset design using nanoparticle experimental properties and anti-glioblastoma drugs from the literature and public databases; (2) Feature engineering by mixing drug assay experimental data with nanoparticle and drug molecular descriptors, resulting in experimental-centered transformation of the original descriptors with the help of the Box-Jenkins moving average operators; (3) Model dataset design by using the new descriptors for pairs of nanoparticles and drugs; (4) Dataset preprocessing (cleaning, standardization, elimination of low variance features); (5) Building of baseline models with ten machine learning methods, using default parameters; (6) Parameter optimization for the best model; (7) Feature selection by eliminating the less important features to obtain the final classification model.

In the case of the drugs, after filtering the dataset, three types of biological activities were considered (vij): EC50, IC50, and LC50. EC50 represents the drug concentration that gives a half-maximal response. IC50 is the concentration of an inhibitor where the response (or binding) is reduced by half. LC50 represents the compound concentration that is lethal for 50% of the population exposed. The natural logarithm was used to transform these values, log(vij). A cutoff value of 10 was used for all activities (close to the mean values). For the nanoparticles, five activities were used as natural logarithm: CC50, EC50, IC50, LC50, and TC50. CC50 represents the 50% cytotoxic concentration defined as the compound concentration that reduced cell viability by 50%, and TC50 defines the compound concentration required to obtain no more than a perceptible effect on 50% of the exposed population of cells. The transformation of these values in a class used a cutoff = 6. All the calculations can be reproduced with the scripts included in our GitHub repository.

Different experimental conditions have been used for:

- Drugs (d): c0 = Biological activity; c1 = cell name, c2 = organism, c3 = target type, c4 = assay organism, c5 = target mapping, c6 = level of confidence, c7 = type of curation, and c8 = assay type;
- Nanoparticles (np): c0(np) = Parameter np assay, c1(np) = Cell line np assay, c2(np) = np shape, c3(np) = np medium, c4(np) = np assay time, c5(np) = surface coating.

Additional information about the mean values used to calculate the final features is presented as Supplementary Materials (SM1-ExperimentalCondition_Means.xlsx).

The original molecular descriptors are different for drugs and nanoparticles:

- For drugs: PSA and ALOGP;
- For nanoparticles: NMUnp, Lnp, Vnpu, Enpu, Pnpu, Uccoat, Uicoat, Hycoat, AMRcoat, TPSA(NO)coat, TPSA(Tot)coat, ALOGPcoat, ALOGP2coat, SAtotcoat, SAacccoat, SAdoncoat, Vxcoat, Vvdw, MGcoat, Vvdw, ZAZcoat, PDIcoat.

np = nanoparticle; npu = nanoparticle elemental unit (Al, SiO_2_, etc.); NMU = number of monomeric units in the np; V = <V(cm^3^/mol)> = average of atomic Van der Waal volume for all atoms in the npu; E = electronegativity; P(A3) = atomic polarizability; L = np large (experimental data); null = not applicable or not available; UC = uncoated nanoparticles; NMU = number of monomer units; HMT = hexamethylenetetramine; TMAOH = tetramethylammonium hydroxide; DMEM = Dulbecco’s modified Eagle’s medium; coat = np coating; Uc = unsaturation count; Ui = unsaturation index; Hy = hydrophilic factor; AMR = Ghose-Crippen molar refractivity; TPSA(NO) = topological polar surface area using N, O polar contributions; TPSA(Tot) = topological polar surface area using N, O, S, P polar contributions; ALOGP2 = squared Ghose-Crippen octanol/water partition coefficient (logP^2^); SAtot = total surface area from P_VSA-like descriptors; SAacc = surface area of acceptor atoms from P_VSA-like descriptors; SAdon = surface area of donor atoms from P_VSA-like descriptors; Vx = McGowan volume; VvdwMG = van der Waals volume from McGowan volume; VvdwZAZ = van der Waals volume from Zhao-Abraham-Zissimos equation; PDI = packing density index.

The final features, such as experimental descriptors, DXj(ci), were obtained by the difference (D) between the original descriptor (Xj) and the mean of the descriptor under specific experimental conditions (ci): DXj(ci) = Xj—mean(X)ci. The name of the model features will have the format [d_/np_] [original descriptor name]([experimental condition]). For example:

- d_DPSA(c1) = difference (D) between original values of PSA descriptor and the mean of PSA values in experimental condition c1 (for drugs, d_);
- np_DLnp(c5) = difference between original L value and the mean of L values in experimental condition c5 (for nanoparticles, np_/np).

An extra input feature (probability) was created as the probability of c0 for drug-NP pairs (count of the number of drug-NP pairs for each c0 activity type/total number of pairs). The final output variable (Class) was calculated using drug and nanoparticle desirability depending on the biological activity:

- For drugs: priori desirability was −1 for EC50 and IC50, and 1 for LC50;
- For NPs: priori desirability was −1 EC50 and IC50, and 1 for CC50, LC50, TC50.

New temporal columns have been created for the Good/Bad classes for drugs and NPs: ‘Good’ if desirability = 1 and log(vij) > cutoff or desirability = −1 and log(vij) < cutoff; ‘Bad’ if desirability = 1 and log(vij) < cutoff or desirability = −1 and log(vij) > cutoff. The final output variable (Class) has a value of 1 if both columns for drug and NP are ‘Good’ (otherwise, it has a value of 0). The initial dataset with drug-NP pairs has 855,129 instances and 119 input features. The input feature values were standardized. All the scripts for obtaining the final dataset and the raw datasets can be found in the GitHub repository: https://github.com/muntisa/nano-drugs-for-glioblastoma (accessed on 21 October 2021). The raw datasets with drug and nanoparticle descriptors and other descriptions from public datasets and the literature can be downloaded from the same repository. Thus, the raw drug descriptors from drug(neuro).csv as datasets/drug(neuro).zip and the raw nanoparticle descriptors from nano(neuro).csv as nano(neuro).zip have been combined to create drug-nanoparticle pairs of descriptors using the script 0-CreateDatasetWithPairs.ipynb.

Ten Machine Learning scikit-learn classifiers were tested to find the best classifier for the prediction of the desirability of nanodrug carriers in glioblastoma:

1. KNeighborsClassifier = KNN—k-nearest neighbors: It is one of the most popular non-parametric classifiers available. It works by assigning an unclassified sample to the same class as the nearest k samples found in the training set [31].
2. GaussianNB = Gaussian Naive Bayes: It is a simple classification algorithm that is based on Bayes’ theorem, which describes the probability of an event based on prior knowledge of conditions related to said event. It is the simplest and the most popular of all similar classifiers [32].
3. LinearDiscriminantAnalysis = LDA—linear discriminant analysis [33]: It is a supervised statistical method based on the projection of data to a lower dimension. The objective is to maximize the scatter between classes versus the scatter within each class. Thanks to this projection, the task of separating the data should be made easier.
4. LogisticRegression = LR—Logistic regression [34]: It is a linear model with the capacity to estimate the probability of a binary response using different factors.
5. DecisionTreeClassifier = DT—Decision Tree (DT): It a classifier that builds a series of models in the form of a tree structure. Then, it infers its decision rules from the features of said trees. Thus, the paths from root to leaf represent classification rules [35].
6. RandomForestClassifier = RF—Random forest [36]: It consists of a large number of individual decision trees that work as an ensemble. Each individual tree in the random forest makes a prediction, and then, the class with the largest amount of votes is chosen as the model’s prediction. Each tree is generated using a bootstrap sample drawn randomly from the original dataset using a classification or regression tree (CART) method and the Decrease Gini Impurity (DGI) as the splitting criterion [36]. RF is mainly characterized by low bias, low correlation between individual trees, and high variance.
7. XGBClassifier = XGB—XGBoost: It is a tree-based ensemble method in which weak classifiers are added in order to correct errors (sequential trees [37]). It should be noted that this classifier demonstrates an excellent performance through the Kaggle competition projects [38].
8. GradientBoostingClassifier = Gradient Boosting for classification—GB classifier: Gradient Boosting is a technique that produces a prediction based on an ensemble of weak prediction models (in general, decision trees) [39].
9. BaggingClassifier = Bagging classifier: Similarly to a GB classifier, a Bagging classifier is an ensemble meta-estimator, meaning that it uses as a basis a number of weaker prediction models in order to make its own prediction. It fits each base classifier on a random subset of the original dataset and then aggregates all the individual performances in order to form a final prediction [36].
10. AdaBoostClassifier = AdaBoost classifier: In a similar fashion to the two previous examples, an AdaBoost classifier is a meta-estimator that first fits a classifier on the original dataset and, subsequently, fits a series of copies of said classifier on the same dataset but adjusting the weights of incorrectly classified instances, meaning that the following classifiers will focus on the most difficult cases [36].

The entire processing of the dataset and ML was done using scikit-learn from python in Jupyter notebooks (see GitHub repository). In the first step, the initial dataset was divided into 75% training and 25% test subsets (using stratification = maintain the same ratio of positive and negative classes in each subset). Thus, the training/test subsets have 641,346/213,783 instances. Based on the training subset, the initial number of features of 119 was lowered to 104 by removing the features with a variance of less than 0.0001. The following features were removed: np_DVxcoat(c5), np_DPDIcoat(c5), np_DHycoat(c5), np_DTPSA(Tot)coat(c5), np_DAMRcoat(c5), np_DSAacccoat(c5), np_DALOGP2coat(c5), np_DUccoat(c5), np_DVvdwMGcoat(c5), np_DSAdoncoat(c5), np_DUicoat(c5), np_DVvdwZAZcoat(c5), np_DALOGPcoat(c5), and np_DSAtotcoat(c5). These are nanoparticle descriptors for experimental condition c5. The featured data for the resulting subsets were standardized in order to speed up future ML methods.

A baseline calculation was done using ten ML methods: KNN, GaussianNB, LDA, LR, DT, RF (100 estimators), XGB (100 estimators), GB, Bagging, and AdaBoost. The calculated metrics were accuracy (ACC), area under the receiver operating characteristics curve (AUROC) [40,41], precision, recall, and f1-score [42]—script 1_CreateDataSets_BaselineML.ipynb.

With the best ML method from the baseline results, new improvements have been made by using a search grid for the best hyperparameters of the best classifier (number of decision trees and number of instances to use in each tree)—scripts 2-Grid Search.ipynb, 2-Grid Search2.ipynb, and 2-Grid Search3.ipynb. In the next step, a feature selection was applied to reduce the number of features by using the feature importance for the best classifier (3-BestModel.ipynb). In the case of the ensemble methods using decision trees, the feature importance was calculated as the mean of feature importance in all decision trees (sklearn function). The feature importance values were normalized to values between 1 and 0 and only features with importance greater than 10% were maintained into the final dataset with the best classifier.

## 5. Conclusions

The current PTML models combine drug and nanoparticle descriptors with the experimental conditions into the perturbation of molecular descriptors for the prediction of anti-glioblastoma nanodrug carriers. The best classification model is based on 41 selected features for 855,129 drug-nanoparticle complexes, a Bagging classifier with 20 decision trees, AUROC of 0.96, and accuracy of 87%. The model could be used to virtually screen a huge number of possible nanoparticle-drug complexes for anti-glioblastoma activity. This could be useful in further studies in search of a less invasive treatment for this disease. All the calculations can be reproduced using the GitHub repository available at https://github.com/muntisa/nano-drugs-for-glioblastoma (accessed on 21 October 2021).

## Figures and Tables

**Table 1:** Baseline classification models for drug-decorated nanoparticle delivery systems against glioblastoma. (We included bold letters to help readers to locate the best values).

Method	ACC	AUROC	Precision	Recall	f1-Score
KNeighborsClassifier	0.7093	0.7882	0.7121	0.7093	0.7105
GaussianNB	0.6553	0.6752	0.6203	0.6553	0.5968
LinearDiscriminantAnalysis	0.7266	0.7988	0.7220	0.7266	0.7236
LogisticRegression	0.7206	0.8002	0.7150	0.7206	0.7169
DecisionTreeClassifier	0.8586	0.8544	0.8576	0.8586	0.8580
RandomForestClassifier	0.7923	0.8714	0.7943	0.7923	0.7931
XGBClassifier	0.7574	0.8502	0.7566	0.7574	0.7570
GradientBoostingClassifier	0.7599	0.8526	0.7603	0.7599	0.7601
BaggingClassifier	**0.8657**	**0.9475**	**0.8655**	**0.8657**	**0.8656**
AdaBoostClassifier	0.7175	0.8100	0.7100	0.7175	0.7119

## Supplementary Materials

The following are available online at https://www.mdpi.com/article/10.3390/ijms222111519/s1.
